# The Biopsychosocial Health Model Differentiates Long‐Term Exercisers From Non‐Exercisers: A Cross‐Sectional Study

**DOI:** 10.1002/hsr2.70476

**Published:** 2025-02-19

**Authors:** Liza Komáromi, Angéla Somogyi, Attila Szabo

**Affiliations:** ^1^ Doctoral School of Education, Faculty of Education and Psychology ELTE Eötvös Loránd University Budapest Hungary; ^2^ Faculty of Health and Sport Sciences Széchenyi István University Győr Hungary; ^3^ Institute of Health Promotion and Sports Sciences, Faculty of Education and Psychology ELTE Eötvös Loránd University Budapest Hungary

**Keywords:** exercise, health, physical activity, sedentary, well‐being

## Abstract

**Background and Aims:**

The biopsychosocial (BPS) model considers that health and behavioral outcomes result from the interaction of biological, psychological, and social factors. Regular exercise is an essential component of modern‐day healthy living. All three factors in the BPS model are related to long‐term exercise. Therefore, this cross‐sectional study aimed to compare adults exercising regularly for at least 3 years with their non‐exercising counterparts on perceived health and stress (antagonistic, biopsychological), life satisfaction (psychological), and perceived income (psychosocial) while controlling for age, gender, and education level.

**Methods:**

Participants were 461 Hungarian volunteers (74.83% female) aged 18−73 years. There were 274 regular exercisers and 187 non‐exercisers. They completed the study on the Qualtrics research platform.

**Results:**

A statistically significant (*p* < 0.001) multivariate analysis of covariance revealed that long‐term exercisers scored lower on perceived stress and higher on perceived health, income, and life satisfaction than non‐exercising adults. Consequently, the three factors of the BPS model differentiated the groups. Still, the effect sizes were relatively small. Finally, perceived stress was a statistically significant (*p* < 0.001) mediator of life satisfaction.

**Conclusion:**

These findings support the idea that long‐term exercise behavior can be studied via the BPS model and the role of stress in life satisfaction. The results have practical implications for promoting a healthy and satisfactory life targeting multi‐level development through exercise based on the BPS model.

## 1 Introduction

The biopsychosocial model (BPS) [1] examines how biological, psychological, and social factors influence health and behavior. The BPS model's holistic approach allows for more comprehensive studies and interventions that address the multifaceted nature of health and well‐being. The model considers the complex interplay between psychobiological factors like health appraisal and stress experience, psychological factors like life satisfaction, and social factors like income. Considering the BPS model, people who incorporate physical exercise into their lifestyle accrue advantages over inactive or non‐exercising individuals [2]. It was recently suggested that “The complex and dynamic nature of physical activity behavior requires a transdisciplinary perspective integrating the interplay of biological, psychological, and social factors” [3].

The BPS model could account for the benefits of regular exercise [4]. Recent reviews highlight several of its bio‐psychophysiological advantages, with Mandolesi et al. [5] reporting biological and psychological gains. Social benefits are also evident, although team‐based exercises may yield more advantages [6]. In clinical settings, the BPS seems effective in exercise programs for patients with chronic lower back pain [7] and in cognitive exercise therapy approach used in improving health parameters in systemic sclerosis patients [8].

**Figure 1:**
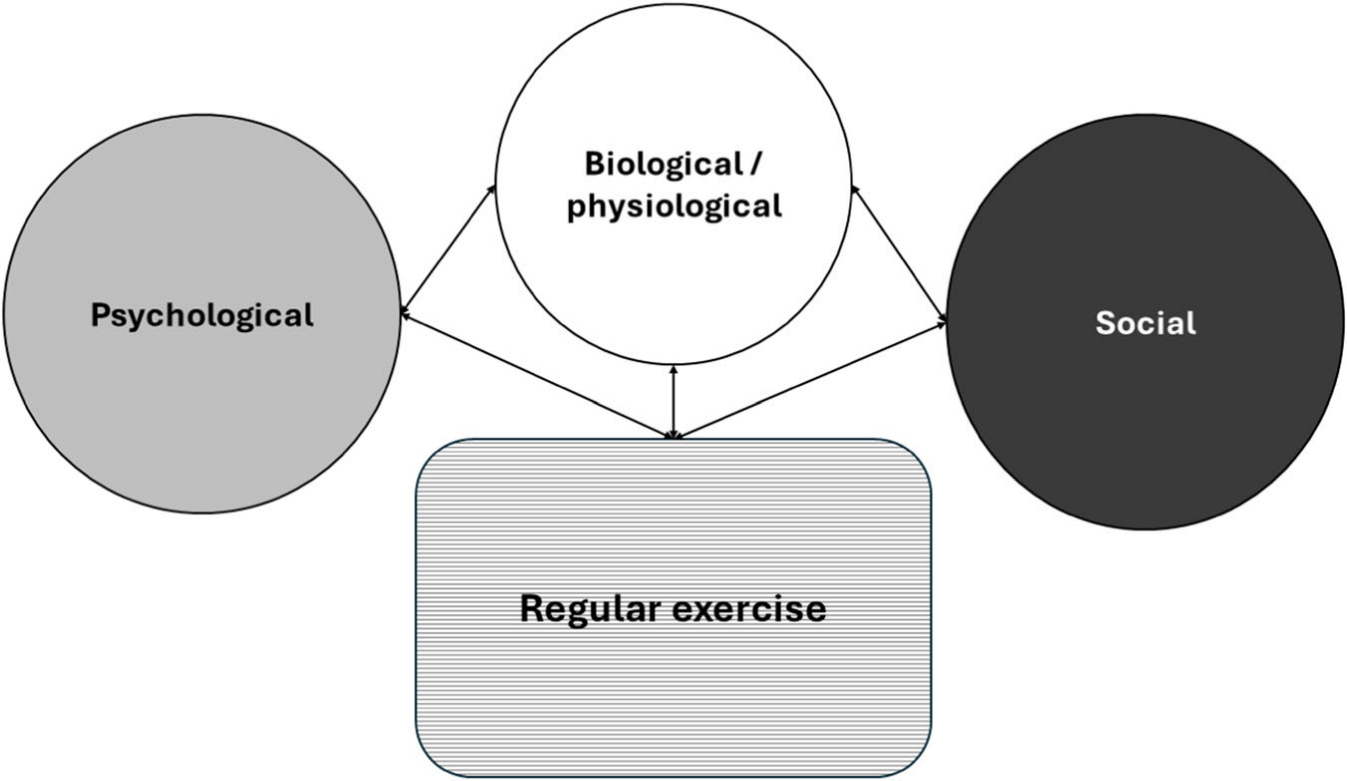
The biopsychosocial (BPS) model and its interactions with long‐term exercise.

Furthermore, regular exercise correlates with income, as Hyytinen and Lahtonen [9] found that exercising men earned 14%–17% more over time. However, Cialani and Mortazavi [10] suggested that perceived income impacts health more than actual income. These findings affirm the interplay of physical, psychological, and social factors in exercise behavior, as reflected in the BPS model (see Figure 1).

The benefits of regular exercise under the BPS model are most likely to manifest in the long term, as interrupted exercise patterns may not yield the same advantages [11]. However, there is no consensus on what constitutes long‐term exercise. Definitions range from 1 year [12] to 4 years [13], with some considering as little as 24 weeks sufficient [14]. Based on existing literature, 2–3 years of uninterrupted regular exercise may meet the criteria. For this study, we adopt a conservative definition, classifying long‐term exercise as consistent weekly activity over 3 years, indicating it has become a lifestyle.

### 1.1 Physical Exercise

Physical exercise is associated with perceived stress and life satisfaction [15]. A study involving 199 adult women revealed that a 10‐week recreational exercise led to a 33.57% reduction in stress levels and a 14.39% increase in life satisfaction [16]. Research has shown that regular exercise lowers health risks and enhances life satisfaction in adults [17] and university employees [18]. A meta‐analysis further supports these findings [19].

### 1.2 Psychobiological Indices in the BPS Model: Perceived Health and Perceived Stress

Perceived health relates to physical symptoms [20] and illness [21]. Perceived stress is linked to gastrointestinal symptoms [22], disturbed sleep, increased cardiovascular risk [23], and depression [24]. Therefore, the subjective appraisal of health and stress can be perceived as a reflection of biological/physiological functions in the BPS model. Exercising individuals perceive better health than non‐exercisers, as shown in a 17‐nation European study of 16,230 participants [25] and a study of 163,131 adults, which also highlighted variations by exercise type and frequency [26]. These findings reflect the psychobiological link between subjective appraisal and physiological functions.

### 1.3 Psychological Index in the BPS Model: Life Satisfaction

Psychobiological factors under the BPS model are positively related to life satisfaction through perceived health [27] and negatively related to mental stress [28]. For example, a study of 2925 Chinese medical students found a moderate negative correlation (*r* = −0.35) between stress and life satisfaction [29]. Therefore, psychological indices, like life satisfaction, are closely linked to the other components of the BPS model.

### 1.4 Social Index in the BPS Model: Perceived Income

Income is also linked to biopsychological markers like stress levels [30] and cognitive appraisal of life satisfaction [31]. Additionally, income seems to have an inverse relationship with subjectively perceived health [32]. An early study suggests this relationship might be directional, with income changes preceding changes in health [33]. However, recent findings point to the primary importance of perceived income in contrast to actual income [10]. Moreover, further supporting the interaction between the BPS model and exercise, a higher income has been associated with increased self‐reported exercise levels [34, 35].

### 1.5 Objectives and Hypotheses

This study aimed to assess differences in mental health indices aligned with the BPS model between long‐term exercisers and non‐exercising adults. We focused on the former group because they likely integrated exercise into their lifestyle, gaining substantial experience and associated benefits. We hypothesized that long‐term exercisers would exhibit better mental health across four BPS model indices than non‐exercisers. Additionally, based on past research, we expected stress to mediate the link between exercise status and life satisfaction.

## 2 Methods

### 2.1 Sample Size Calculation

We utilized G*Power (v.3) software [36] to calculate the minimum sample size necessary for our statistical analyses. For a multivariate analysis of variance (MANOVA), we aimed to detect main effects and interactions with a statistical power of 0.95, a medium effect size (*V*) of 0.0625, and a significance level (*α*) of 0.05 while accounting for two groups, maximum seven predictors (including potential covariates), and four dependent measures. This calculation indicated a minimum sample size of 144 participants. Additionally, for Chi‐square (*χ*²) tests with 2 degrees of freedom, a power of 0.95, and a medium effect size (*φ*) of 0.30, the minimum required sample size was determined to be 172. Our final sample included 461 participants, far exceeding the calculated minimum values.

### 2.2 Ethical Permission

This cross‐sectional study was conducted anonymously, ensuring researchers could not identify any participants. The Research Ethics Committee of the Faculty of Education and Psychology at ELTE Eötvös Loránd University granted ethical approval (Certificate No. 2022/517). The study adhered to the principles outlined in the Helsinki Declaration (World Medical Association 2013), and we followed the guidelines of the British Psychological Society for Internet‐based research [37]. Informed consent was obtained from all participants before their involvement in the study.

### 2.3 Participants

We primarily recruited participants for this study through social media platforms such as Twitter, Facebook, Instagram, and LinkedIn and supplemented it with the snowball method to boost the sample size. Eligible participants were individuals aged 18 years or older who exercised continuously for at least 3 years or not at all. We kept the eligibility criteria minimal to maximize participation and response rates and limited the number of survey questions following De Koning et al. [38] recommendations for increasing online response rates. A pilot test with 12 university students indicated that it took 5−10 min to complete the survey, even by slower readers or those who took more time to consider their answers.

We gathered responses from 556 adults. However, we had to disregard 95 because they reported exercising regularly but had less than 3 years of continuous exercise history. Based on Caspersen et al. [39], we define regular exercise as a weekly planned physical activity requiring skeletal muscle activity and energy expenditure. The final sample comprised 461 participants with a female majority (*n* = 345, 74.83%). Their (M) age was 40.40 years (±SD = 11.61), ranging from 18 to 73. More than half of the sample (59.0%) completed university education. Most respondents exercised regularly (*n* = 274, 59.44%) for 3 or more years, and 187 (40.56%) were non‐exercisers. The exercising individuals reported an average exercise history of 20.89 years (±SD = 13.04) ranging from 3 to 55 years.

### 2.4 Materials

The survey included demographic questions covering age, gender, exercise status, exercise history in years, and education level categorized as primary, secondary, or university. Participants also rated their perceived health status and income on a single‐item 7‐point Likert scale [40], ranging from ‘*very bad*’ to ‘*very good*.’ In addition to the demographic questions, participants completed two widely employed psychometrically validated instruments, detailed below.

#### 2.4.1 Perceived Stress Scale (PSS)

The PSS [41] measures how individuals have perceived stress over the past 2 weeks. The PSS consists of 14 items, with total scores ranging from 0 to 56. Participants rate their perceived stress on a 5‐point Likert scale (0 = *never*; 4 = *very often*). Positive items indicate perceived coping self‐efficacy, while negative items reflect feelings of hopelessness, a significant predictor of depression [42]. Higher PSS scores strongly predict adverse health outcomes [43]. The PSS has demonstrated reliable psychometric properties, with the original scale exhibiting good internal consistency (Cronbach's alpha [*α*] = 0.75) and test−retest reliability ranging from 0.84 to 0.86 over 2 weeks [41]. In this study, we used the Hungarian version, psychometrically validated by Stauder and Konkolÿ Thege [44], with a reported internal reliability of 0.79, which in the current study was even better (*α* = 0.88).

#### 2.4.2 Satisfaction With Life Scale

Participants rated their agreement with the following five statements on a 7‐point scale (1 = *strongly disagree* to 7 = *strongly agree*): (1) “In most ways, my life is close to my ideal”; (2) “The conditions of my life are excellent”; (3) “I am satisfied with my life”; (4) “So far I have gotten the important things I want in life”; and (5) “If I could live my life over, I would change almost nothing.” These statements comprise the Satisfaction with Life Scale (SWLS [45]). Scores were calculated by summing the ratings for each item, resulting in a total ranging from 5 to 35. Diener et al. [45] reported that the scale has good internal reliability (*α* = 0.81). In this study, we used the psychometrically validated Hungarian version of the scale [46], which has good internal reliability (*α* = 0.84). In the current sample, the internal reliability of the scale was 0.89.

### 2.5 Procedure

We conducted the study on the Qualtrics research platform [47] from winter 2022 to autumn 2023. Participants accessed the survey through a hyperlink or QR code that led them to an online informed consent form. The form asked them to confirm their understanding of the research protocol, potential risks, anonymity, confidentiality, and participation criteria by clicking a “yes” button. After providing consent, participants accessed the survey, which required them to answer all questions to progress through the sections. This method could discourage some respondents from completing the survey, but it is convenient for researchers because it ensures that there will be no missing data. After completing the study, participants received a thank you message acknowledging the importance and usefulness of their participation.

### 2.6 Data Analyses

The data are available at the Mendeley repository (DOI 10.17632/vd8wjg8d94.1). First, we assessed the normality of the data distribution by examining skewness and kurtosis. Second, we calculated Pearson correlation coefficients to explore relationships between the variables. We then conducted a multivariate analysis of covariance (MANCOVA) with perceived health, income, perceived stress, and life satisfaction as dependent variables. Exercise status served as the grouping factor, while age, education level, and gender were included as covariates. All tests were two‐sided. Due to the small number of males, which would have reduced cell sizes, we controlled for gender instead of using it as a categorical variable along with exercise status. Finally, we used mediation analysis to test whether stress mediates the relationship between exercise and life satisfaction. For most analyses, we used the SPSS software (v. 29 [48]). However, we performed the mediation analysis with the JASP software (v. 0.16.3.0. [49]).

## 3 Results

The skewness and kurtosis values ranged between −1.00 and +1.00, meeting the normality assumption criterion [50, 51]. Correlation results indicated that most dependent and independent variables were correlated (Table 1). They also indicated that age, gender, and education levels correlate with at least one of the dependent measures; therefore, controlling for their effects in the primary analysis was warranted.

**Table 1:** Correlations between the independent and dependent measures.

		PH	PI	PS	LS	EN	MF	Age
PI	*r*	0.275	—					
	*p*	< 0.001						
PS	*r*	−0.167	−0.184	—				
	*p*	< 0.001	< 0.001					
LS	*r*	0.253	0.247	−0.473	—			
	*p*	< 0.001	< 0.001	< 0.001				
EN	*r*	0.361	0.189	−0.156	0.225	—		
	*p*	< 0.001	< 0.001	0.001	< 0.001			
MF	*r*	−0.105	−0.164	0.161	−0.074	−0.153	—	
	*p*	0.024	< 0.001	0.001	0.113	0.001		
Age	*r*	−0.229	−0.037	−0.134	−0.025	−0.092	0.087	
	*p*	< 0.001	0.428	0.004	0.598	0.049	0.061	
ED	*r*	0.142	0.148	−0.032	0.067	0.195	0.137	−0.118
	*p*	0.002	0.001	0.500	0.149	< 0.001	0.003	0.012

Abbreviations: ED = education level, EN = exercise for ≥ 3 years or no exercise, LS = life satisfaction, MF = male or female, PH = perceived health, PI = perceived income, PS = perceived stress.

Consequently, in the MANCOVA, we controlled age, gender, and education level. The MANCOVA was statistically significant for exercise and no‐exercise groups (Pillai's trace = 0.124, *F* (4, 452) = 16.03, *p* < 0.001, effect size [partial Eta squared, *η*_*p*_^2^] = 0.124). Age (Pillai's trace = 0.080, *F* (4, 452) = 9.80, *p* ≤ 0.001, *η*_*p*_^*2*^ = 0.080), gender (Pillai's trace = 0.048, *F* (4, 452) = 5.65, *p* ≤ 0.001, *η*_*p*_^2^ = 0.048), and education level (Pillai's trace = 0.022, *F* (4, 452) = 2.57, *p* = 0.04, *η*_*p*_^2^ = 0.022) were statistically significant covariates. Univariate tests revealed that exercisers differed statistically significantly from the non‐exercising group in all four outcome measurements (Table 2).

**Table 2:** Results of the univariate tests of the differences in four outcome measures between those who exercised at least for 3 years and non‐exercising participants.

Measure	Exercisers mean ± (SD)	Non‐exercisers mean ± (SD)	*F #*	*p*	*η*_*p*_^2^
Perceived health^c^ (range 1 to 7)	5.70 (0.91)	4.95 (1.02)	61.18	< 0.001	0.118
Perceived income^b^ (range 1 to 7)	3.92 (0.92)	3.57 (0.85)	14.03	< 0.001	0.030
Perceived stress^a^ (range 0 to 56)	23.20 (8.27)	25.84 (8.18)	7.61	0.006	0.016
Life satisfaction (range 5 to 35)	25.58 (5.10)	22.90 (6.53)	19.51	< 0.001	0.041

a Gender and age were statistically significant covariates.

b Gender was a statistically significant covariate.

c Age was a statistically significant covariate; # degrees of freedom (*df*) = 1456.

**Figure 2:**
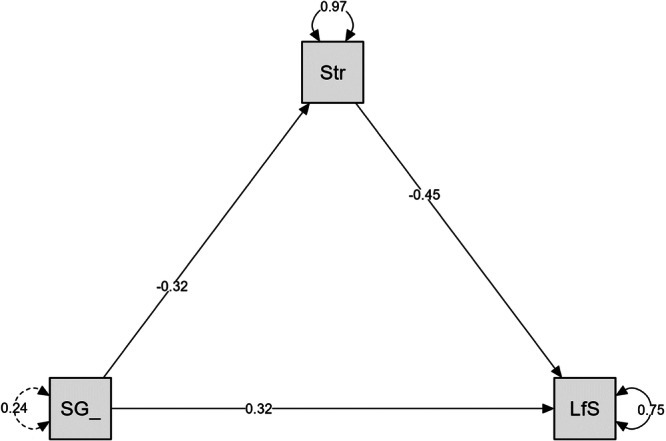
The mediating effect of perceived stress in the relationship between long‐term exercise status and life satisfaction. LfS = life satisfaction, SG = exercise group, Str = stress.

The mediation analysis resulted in statistically significant direct, indirect, and total effects (all *p* < 0.001; *R* ^2^ = 0.247), indicating that long‐term exercise status directly affects life satisfaction and indirectly through perceived stress. The mediation analysis results are summarized in Table 3 and Figure 2.

**Table 3:** Test of the mediatory effects of perceived stress between exercise status and life satisfaction.

Parameter estimates										
									95% confidence interval	
					B	SE	*z‐*value	*p*	Lower	Upper
**a. Direct effects**										
SportGroup_1no_2yes			→	Life satisfaction	0.315	0.08	3.79	< 0.001	0.152	0.478
*Note:* Delta method standard errors, normal theory confidence intervals, ML estimator; SportGroup_1no_2yes = long‐term or no‐exercise group.										
**b. Indirect effects**										
SportGroup_1no_2yes	→	Stress	→	Life satisfaction	0.143	0.04	3.24	0.001	0.056	0.229
*Note:* Delta method standard errors, normal theory confidence intervals, ML estimator.										
**c. Total effects**										
SportGroup_1no_2yes			→	Life satisfaction	0.458	0.09	4.96	< 0.001	0.277	0.639
*Note:* Delta method standard errors, normal theory confidence intervals, ML estimator.										
**d. Path coefficients**								
Stress			→	Life satisfaction	−0.449	0.04	−10.97	< 0.001	−0.529	−0.369
SportGroup_1no_2yes			→	Life satisfaction	0.315	0.08	3.79	< 0.001	0.152	0.478
SportGroup_1no_2yes			→	Stress	−0.318	0.09	−3.39	< 0.001	−0.501	−0.134
*Note:* Delta method standard errors, normal theory confidence intervals, ML estimator.										

## 4 Discussion

This study's novel contribution is the evidence of the BPS model's utility in studying mental health indices associated with long‐term exercise behavior. Although our work cannot be seen as transdisciplinary [3], the factors examined as outcome measures related to the BPS model are strongly associated with biological, psychological, and social factors.

As already discussed, both perceived health and stress are closely associated with numerous symptoms and diseases [20, 21, 22, 23, 24], and therefore, they are an acceptable reflection of the biological and/or physiological factors in the BPS model. Life satisfaction is generally studied as a mental health or psychological measure, not independent of life events, thus adequately representing the psychological factor in the BPS model. Further, perceived income is mainly a psychological or cognitive construct. It cannot be separated from the social‐financial status that is cognitively evaluated compared to others' living standards and wealth. Furthermore, it might significantly impact health more than objective income [10] while positively related to self‐reported exercise levels [34, 35]. While these arguments are empirically supported, future work testing the model must be more transdisciplinary, as John et al. [3] suggested, and in addition to psychological measures, employ objective biological and social markers, which were only indirectly measured in the current research.

Nevertheless, we also assessed education level, considered an objective social index of health [52]. This categorical measure also significantly differentiated long‐term exercisers from non‐exercisers in that completed university education was more frequent (> 20%) in the former group. Therefore, both perceived income as an indirect index and education level as a direct measure of the social factors in the BPS model were more favorable in the exercise than in the comparison group. Although little research has been done in this area, our findings align with two recent reports disclosing a positive connection between regular exercise and education level [53, 54].

Examining the correlation results (Table 1), we found that our results support the model in Figure 1. For example, long‐term exercise correlated most strongly (in order) with perceived health (indirect index of biological factors in the BPS model), life satisfaction (psychological factor in the BPS model), and education level and perceived income (social factors in the BPS model). Therefore, the model depicted in Figure 1 is supported by the results.

Further, disregarding exercise status, correlations between factor variables supported the model. Perceived health was positively, whereas perceived stress was negatively associated with perceived income, supporting the connection between indirect biological factor indices and social factors and earlier research results [20, 21, 22, 23, 24]. This factor connection was further supported by the positive correlation between perceived health and education level, matching past reports [52, 55]. The positive correlation between life satisfaction and perceived income supported the relationship between psychological and social factors in the BPS model, which is also in accord with past reports [31, 56]. However, in the current study, education level did not correlate statistically significantly with life satisfaction. While these findings disagree with some reports in the literature (i.e., [57]), they agree with another work [58] using a 3‐category education assessment as we did in the current study. However, the relationship between education and life satisfaction may not be direct, as shown by an Australian study [59] in which income and health mediated this relationship,^1^ while the direct effect of education on life satisfaction was negative. Finally, we found a positive correlation between life satisfaction and perceived health and a significant negative correlation between life satisfaction and stress, which agree with past reports [27, 28] and support the connection between biological and psychological factors in the BPS model. In brief, the current results lend support for the BPS model.

The negative relationship between perceived stress and life satisfaction also emerged in the mediation analysis regarding the association between long‐term exercise status and life satisfaction. Indeed, perceived stress mediated this relationship, as indicated by the statistically significant indirect effect, but long‐term exercise also directly affected reported life satisfaction, supported by the statistically significant direct effect. Notably, the beta was small to medium, suggesting a moderate role of perceived stress in the relationship between exercise status and life satisfaction. While limited research specifically explored the mediating effect of stress in the exercise−life satisfaction relationship, a recent investigation showed that extracurricular sports and exercise positively impacted life satisfaction, and academic stress was a mediator of the relationship in a sample of Chinese adolescents [60].

### 4.1 Limitations

This study has limitations that should be considered when interpreting the findings. First, the volunteer sample does not represent the general population, and self‐selection could distort the results. Second, causation cannot be implied from this cross‐sectional work. Third, the primarily female sample also calls for a cautious interpretation of the results and replication of the work with a more balanced gender distribution in the sample. Fourth, our conceptualization of long‐term exercise is arbitrary, and it is based on limited research in the literature. Fifth, since we studied a Hungarian sample, the cross‐cultural validity of the study cannot be assured. Finally, based on past research, biological and social measurements mirror biological and social factors but are still subjective appraisals subject to cognitive and memory biases. Future studies should use objective health and social measures, such as aerobic fitness and social class.

## 5 Study Contribution and Conclusion

This study suggests that factors directly or indirectly fitting into the BPS model differentiate long‐term exercisers from non‐exercisers, supporting our hypothesis. However, the effect sizes were small. The relationship between the dependent measures supported the BPS model even without considering exercise status. Perceived stress was a statistically significant yet moderate mediator of the exercise−life satisfaction relationship. Most findings support the extant literature and expand it by demonstrating that indirect indices of biological factors, direct and indirect indices of social factors, and a direct index of psychological factors in the BPS model are linked to prolonged exercise. These findings should be replicated with objective biological measures.

## Author Contributions

**Liza Komáromi:** investigation, validation, methodology, data curation. **Angéla Somogyi:** conceptualization, supervision, visualization, validation, project administration. **Attila Szabo:** writing–original draft, formal analysis, software, validation, methodology, conceptualization. all authors have read and approved the final version of the manuscript.

## Ethics Statement

The Research Ethics Board of the Faculty of Education and Psychology at ELTE Eötvös Loránd University Budapest, Hungary, has approved the study (Permission No. 2022/517).

## Conflicts of Interest

The authors declare no conflicts of interest.

## Transparency Statement

The lead author, Attila Szabo, affirms that this manuscript is an honest, accurate, and transparent account of the study being reported, that no important aspects of the study have been omitted, and that any discrepancies from the study as planned (and if relevant, registered) have been explained.

## Data Availability

Data on which the results of this research are based are available at Mendeley Data Repository (10.17632/vd8wjg8d94.1). Attila Szabo had full access to all of the data in this study and takes complete responsibility for the integrity of the data and the accuracy of the data analysis.

## Endnotes

1 This relationship could also be demonstrated in our data at the Mendeley data repository (DOI 10.17632/vd8wjg8d94.1). However, since this connection was not an objective of this work, we do not report it here.
